# The Effect of Size and Thermal Treatment on the Photoluminescent Properties of Europium-Doped SiO_2_ Nanoparticles Prepared in One Pot by Sol-Gel

**DOI:** 10.3390/ma14071607

**Published:** 2021-03-25

**Authors:** Hussein Fneich, Nathalie Gaumer, Stéphane Chaussedent, Ahmad Mehdi, Wilfried Blanc

**Affiliations:** 1 ICGM, CNRS, ENSCM, University Montpellier, 34095 Montpellier, France; husseinfneich@hotmail.com (H.F.); ahmad.mehdi@umontpellier.fr (A.M.); 2 LPHIA, SFR MATRIX, University of Angers, 49000 Angers, France; nathalie.gaumer@univ-angers.fr (N.G.); stephane.chaussedent@univ-angers.fr (S.C.); 3 INPHYNY, Université Côte d’Azur, CNRS UMR 7010, Parc Valrose, 06108 Nice, France

**Keywords:** luminescent materials, silica nanoparticles, rare-earth ions, europium, sol-gel, photoluminescence

## Abstract

Europium (Eu)-doped silica nanoparticles have attracted great interest for different applications, in particular in biomedicine as biosensors or for tissue regeneration. Sol-gel is the most common process used to prepare those particles, with size varying from tens to hundreds of nanometers. In this article, we focus our attention on the comparison between two commonly used sol-gel derived methods: reverse microemulsion (for particles smaller than 100 nm) and Stöber method (for particles larger than 100 nm). Europium concentration was varied between 0.2 and 1 mol%, and the nanoparticle diameters were 10, 50 and 100 nm. The link between the local environment of europium ions and their optical properties was investigated and discussed. Using Transmission Electron Microscopy, nitrogen sorption, X-ray diffraction, Fourier-Transform Infra-Red and pulsed doubled Nd:YAG laser, we confirmed that fluorescence lifetime was improved by thermal treatment at 900 °C due to the elimination of aqueous environment and modification of structure disorder. The size of nanoparticles, the amount of europium and the thermal treatment of obtained materials influence the emission spectra and the decay curves of Eu^3+^.

## 1. Introduction

For decades, silica glass doped with rare-earth ions has become a material of prime importance to support the science of photonics [1,2,3,4]. The choice of this glass is strategic because it combines the mechanical and chemical advantages of silica, compared to other glasses, and the exceptional spectroscopic properties of rare-earth ions arising from their 4f intra-configurational electronic transitions. By judiciously choosing the appropriate rare-earth ion, it is possible to engineer absorption and emission properties in the UV, visible and infra-red regions [5]. For instance, many studies have been devoted to rare-earth ions such as erbium, ytterbium and thulium ions due to their ability to emit in the low-loss window of silica glass (around 1.5 µm), of interest for fiber lasers and amplifiers [6].

The development of nanotechnology also raises interest in silica-based nanoparticles, in particular for europium-doped silica nanoparticles. Europium ion, in its trivalent state, emits in the red part of the visible spectrum, and it is extensively used for phosphors. This ion is also well known as a structural probe to analyze matrix material structures [7,8]. Indeed, the ^5^D_0_ → ^7^F_2_ transition probability is very sensitive to the crystalline field around this ion, i.e., to relatively small changes in the chemical surroundings of the Eu^3+^ ion. It has been proposed that Eu-doped silica nanoparticles be embedded into polymer fibers for luminescent properties [9]. Over the past two decades, Eu-doped silica nanoparticles have attracted tremendous interest in biology where they can be used as biosensors [10] or for tissue regeneration [11] due to their biocompatibility.

Different fabrication processes have been reported to prepare such Eu-doped silica nanoparticles: microwave assisted combustion [12], mechanochemical solid-state reaction [13] and sol-gel [14,15,16]. The latter is the most common process as it allows the preparation of nanoparticles smaller than 100 nm with the reverse micro emulsion method, and particles larger than 100 nm with the Stöber one. However, despite the interest in these nanoparticles, direct comparison of the structural and optical properties of nanoparticles prepared with both processes is lacking in the literature.

Therefore, we focus this paper on the preparation of silica nanoparticles (SiNP) prepared by the sol-gel route with the reverse micro emulsion and Stöber methods, doped with different molar percentages of europium, as well as the study of their different structural and photoluminescent properties. The europium concentration (0.2, 0.5 and 1 mol%) was chosen in accordance with luminescent and biological applications [13]. Moreover, to study the effect of SiNP size on optical properties, different sizes were prepared and studied (10, 50 and 100 nm). In addition, thermal treatment is usually applied to remove unwanted species. This step may alter the structure of the silica, such as favoring SiO_2_ condensation in a three-dimensional network leading to a more organized structure after decreasing the concentration of silanol groups (Si-OH) [17,18,19,20]. The study of the influence of thermal treatment at 900 °C for 5 h on their different properties is presented and discussed.

## 2. Sample Preparation

Two sol-gel methods were applied to prepare silica nanoparticles named **SiNPX:YEu**, where X is the nanoparticles size and Y is the amount of europium. Their starting compositions and nomenclatures are given in Table 1. Stöber method [21,22] was used to synthesize the larger SiNP (~100 nm, while we used the sol-gel process in reverse microemulsion to prepare the smaller SiNP (10 and 50 nm) based on the works of Yang et al. [23] and Touisni et al. [24], respectively. To study the effect of the thermal treatment, half of the obtained powder of each sample was heated at 900 °C for 5 h using a heating rate of 10 °C/min and named **SiNPX:YEu@900**.

### 2.1. Synthesis of Europium-Doped 10 nm Silica Nanoparticles by Sol-Gel in Reverse Micro-emulsion

The appropriate amounts of EuCl_3_.6H_2_O (99.9%, Sigma-Aldrich, Saint-Quentin-Fallavier, France) were dissolved in 2.7 mL of ultra-pure water. After their total dissolution, 0.9 mL of NH_4_OH 27% and then 46.5 mL of cyclohexane (anhydrous, 99.5%, Sigma-Aldrich, Saint-Quentin-Fallavier, France) were added. Afterwards, 22.5 mmol of tetramethylorthosilicate TMOS (98%, Sigma-Aldrich, Saint-Quentin-Fallavier, France) were added dropwise. The obtained mixture was stirred for 10–15 min at room temperature. Then, 50.45 mL of Triton X-100 (laboratory grade, Sigma-Aldrich, Saint-Quentin-Fallavier, France), 18.8 mL of Hexan-1-ol (anhydrous, ≥99%, Sigma-Aldrich, Saint-Quentin-Fallavier, France) and 12 mL of ultra-pure water were added. The reaction was maintained for 24 h. After that, the solution was centrifuged for 30 min at a speed of 20.000 rpm. The nanoparticles were washed twice, once with a mixture of water and ethanol (1:2) and once with pure ethanol. A second centrifugation took place for 30 min at a speed of 20.000 rpm followed by two washes with pure ethanol. The nanoparticles were heated to 80 °C under air for 24 h to ensure the total evaporation of all solvents. White powders were finally obtained.

### 2.2. Synthesis of Europium-Doped 50 nm Silica Nanoparticles by Sol-Gel in Reverse Micro-emulsion

Micelles were prepared, firstly by mixing 42.4 mL of Triton X-100, 43.2 mL of Hexan-1-ol, 100 mL of cyclohexane and 4.5 mL of ultra-pure water at room temperature. The mixture was stirred for 15 min so that the reverse micelles could form. A transparent solution was finally obtained. The appropriate amounts of EuCl_3_.6H_2_O were dissolved in 4.5 mL of ultra-pure water, then added to the initial mixture and the combination was stirred for 10 min. Next, 21.72 mmol of tetramethylorthosilicate TMOS were added dropwise on the reverse microemulsion. The mixture was stirred vigorously for 15 min to ensure good contact between the TMOS and the aqueous phase in the core of reverse micellar droplets. Then, 3.2 mL of NH_4_OH 27% were added and the reaction was maintained for 24 h. Finally, the same centrifuging, washing and drying protocols described above were applied.

### 2.3. Synthesis of Europium-Doped 100 nm Silica Nanoparticles by Sol-Gel with Stöber Method

The appropriate amounts of EuCl_3_.6H_2_O were dissolved in 36 mL of ultra-pure water. After their total dissolution, 100 mL of methanol were added, followed by 13.9 mL of NH_4_OH 27%. The obtained mixture was stirred for 10–15 min at room temperature. Then, 28 mmol of tetraethylorthosilicate TEOS (99.999%, Sigma-Aldrich, Saint-Quentin-Fallavier, France) were added slowly. TEOS is used for the Stöber method because of its slower initiation reaction than TMOS (used for the reverse micro-emulsion process). The reaction was maintained for 60 min. Finally, the same centrifuging, washing and drying protocols described above were applied.

## 3. Characterization Techniques

Inductively-coupled plasma—optical emission spectrometry (ICP-OES) analysis was carried out using Perkin-Elmer Optima 7000 DV (Waltham, MA, USA), and sample mineralization was performed by wet acid in polytetrafluoroethylene (PTFE) beakers at a temperature between 250 and 280 °C. Transmission electron microscopy (TEM) images were taken using a 1200EX2 electron microscope (JEOL, Tokyo, Japan) at an acceleration tension of 100 kV equipped with an EMSIS camera mounted with an 11-megapixel CCD sensor (EMSIS GmbH, Muenster, Germany). N_2_ Physisorption experiments were carried out at −196 °C on a TriStar 3000 instrument (Micromeritics, Norcross, GA, USA). Samples were outgassed under vacuum at 150 °C overnight. Equivalent BET specific surface areas were determined in the relative pressure range P/P_0_ from 0.01 to 0.3 using 74 points. The total pore volume was measured at P/P_0_ > 0.985. X-ray Diffraction (XRD) experiments were performed using an X’Pert MPD θ-θ diffractometer (Philips, Amsterdam, The Netherlands) with Cu Kα radiation (λ = 1.5418 Å). It was equipped with the X’Celerator detector and nickel filter. Analyses were carried out between 12° and 60° at intervals with a step size of 0.033. Fourier-Transform Infra-Red (FTIR) analysis was carried out in the range 400–4000 cm^−1^ using a Spectrum Two™ spectrometer (Perkin Elmer, Waltham, MA, USA) in Attenuated Total Reflectance (ATR) mode. The spectra were obtained from the accumulation of 10 scans at a resolution of 2 cm^−1^. Elementary analysis was carried out with a Vario Micro Cube (Elementar, Munich, Germany) equipped with a UMX5 Comparator (Mettler Toledo, Zurich, Switzerland) balance with 0.1 µg precision. The samples were combusted at 1150 °C to form an H_2_O gas. The mass percentages measurement of hydrogen was detected by a thermal conductivity detector (TCD) katharometer (Elementar Americas Inc., Ronkonkoma, NY, USA). A pulsed doubled Nd:YAG laser (Spectra-Physics, Santa Clara, CA, USA) emitting at 532 nm with 10 Hz frequency was used as excitation source for the photoluminescence and lifetime measurements of europium. All measurements were performed with a photon-counting device (Stanford Research Systems, Sunnyvale, CA, USA) at room temperature. The decay curves were carried out around the maximum of the emission line of europium, with a temporal window width of 10.24 μs [25].

## 4. Results and Discussion

**Figure 1:**
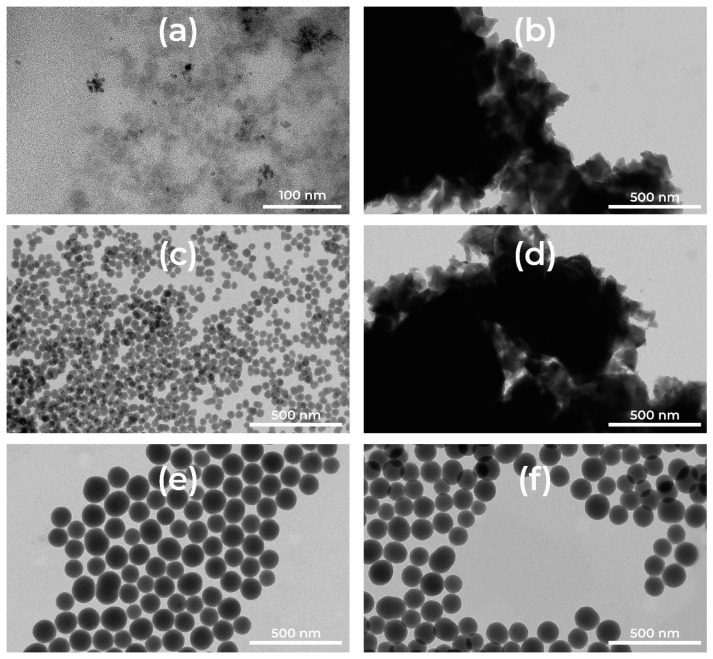
TEM images of: (**a**) SiNP10:1Eu; (**b**) SiNP10:1Eu@900; (**c**) SiNP50:1Eu; (**d**) SiNP50:1Eu@900; (**e**) SiNP100:1Eu; and (**f**) SiNP100:1Eu@900.

Figure 1 shows the TEM images before and after thermal treatment. We present as an example the 1% europium-doped silica nanoparticles (SiNP). The images show dispersed SiNP for all the samples before thermal treatment (Figure 1a,c,e). The latter affects the SiNP of a size less than 50 nm prepared by sol-gel in inverse microemulsion, where the nanoparticles lose their spherical form and coalesce (Figure 1b,d), while SiNP larger than 100 nm prepared by Stöber method keep their spherical shape and size without any coalescence. Surfactants were used during the reverse micro emulsion process to prepare the smallest SiNPs (10 and 50 nm). Those surfactants may react during heat treatment and may favor the melting of SiNPs with size less than 50 nm.

**Figure 2:**
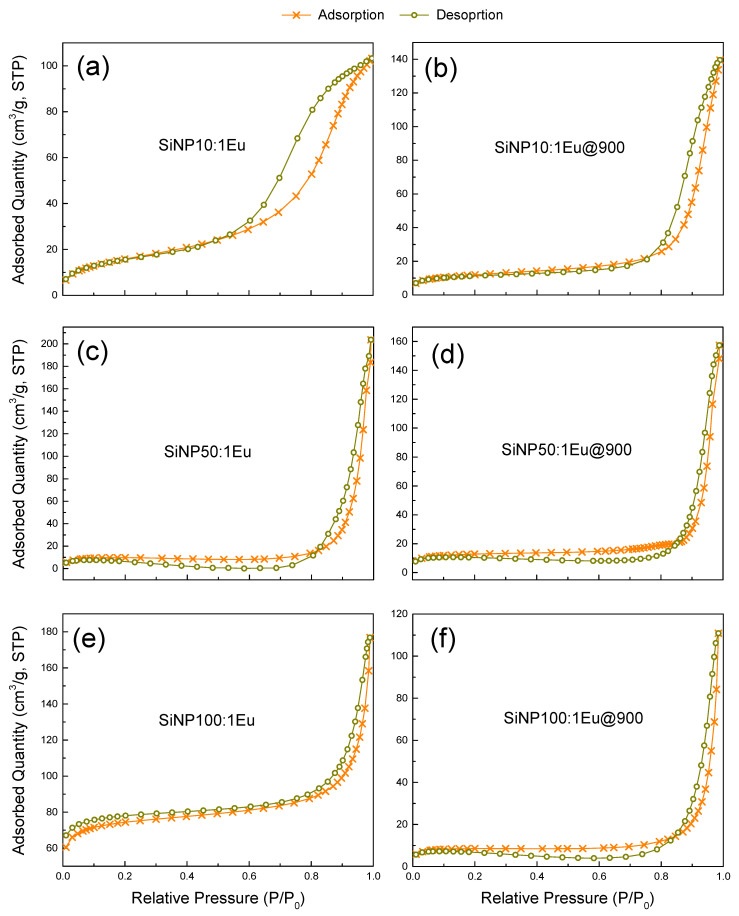
Adsorption-desorption isotherms of all **SiNPX:1Eu** and all **SiNPX:1Eu@900**.

Furthermore, the N_2_ adsorption isotherms of Eu-doped SiNP before thermal treatment are Type II (Figure 2), characteristic of a non-porous or macroporous material, while the isotherms of the materials after thermal treatment are Type III. While these are also characterized by their non-porous or macroporous nature, the two types of isotherms differ in the interactions between the material and the N_2_, which is lower in the case of isotherm Type III [26]. In addition, the 1% europium-doped SiNP shown are representative. We note that the thermal treatment increased the adsorbed quantity of the N_2_ for the 10 nm SiNP, whereas it was reduced for the SiNP of sizes close to 50 nm and greater than 100 nm, which is related to the inter-grain porosity. Table 2 shows the surface areas measured by the BET method and the pore volumes of the 1% Eu-doped SiNP and, for comparison, those obtained for the 1% Eu-doped silica bulks studied and discussed earlier [25]. Before thermal treatment, the surface area of the SiNP of 10 and 50 nm sizes was lower than with 100 nm SiNP, probably due to a higher agglomeration rate of the smallest particles.

In comparison with the Eu-doped silica bulk, the thermal treatment induces the same effect on the surface areas of the nanoparticles. For all silica nanoparticles, the thermal treatment results in a reduction of the surface area of 30, 25 and 89% for **SiNP10:1Eu**, **SiNP50:1Eu** and **SiNP100:1Eu**, respectively, which follows a similar line to the densification observed by TEM for **SiNP10:1Eu** and **SiNP50:1Eu.** The large reduction of the surface area of **SiNP100:1Eu** particles is attributed to their preparation with the Stöber method which leads to softer particles. On the other hand, the pore volume decreases after thermal treatment for all samples except for **SiNP10:1Eu**, where it increases, which explains the difference for adsorbed N_2_ observed in the isotherms: it increases for **SiNP10:1Eu** and decreases for the others.

**Figure 3:**
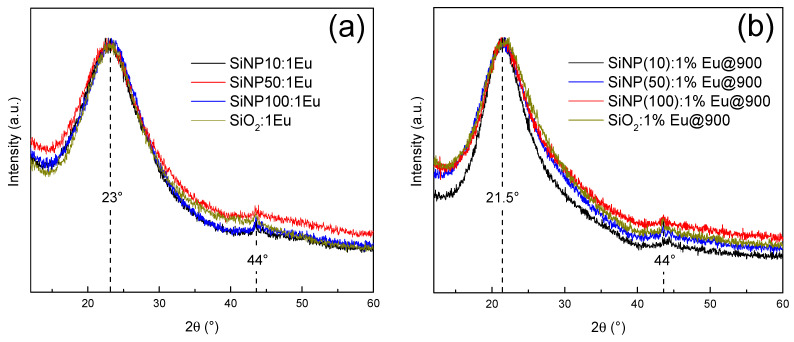
XRD patterns of **SiNPX:1Eu** and of bulk **SiO_2_:1Eu**: (**a**) before thermal treatment; and (**b**) after thermal treatment.

Figure 3 shows the XRD patterns of 1% Eu-doped silica nanoparticles before and after thermal treatment. For all the SiNP samples, the XRD patterns exhibit the same behavior as those obtained for the europium-doped silica bulk. The peak is always the same, at 2θ ~44°, corresponding to an artifact due to the sample holder used, as well as the wide band at 2θ ~ 23° corresponding to the signature of the amorphous structure of the network [19,27]. However, thermal treatment does not cause crystallization of any sample. There is no presence of peaks corresponding to Eu_2_O_3_ crystals, normally represented as an intense diffraction peak around 2θ = 29° and two less intense peaks around 2θ = 33° and 47° [28,29]. This observation is consistent with a good dilution of europium ions in the SiNP. We also note the offset of the position of the wide band towards lower 2θ (from 21.5° to 23°) which corresponds to the decrease in the disorder of the amorphous structure [19], also confirmed by a decrease in the full width at half maximum (FWHM).

**Figure 4:**
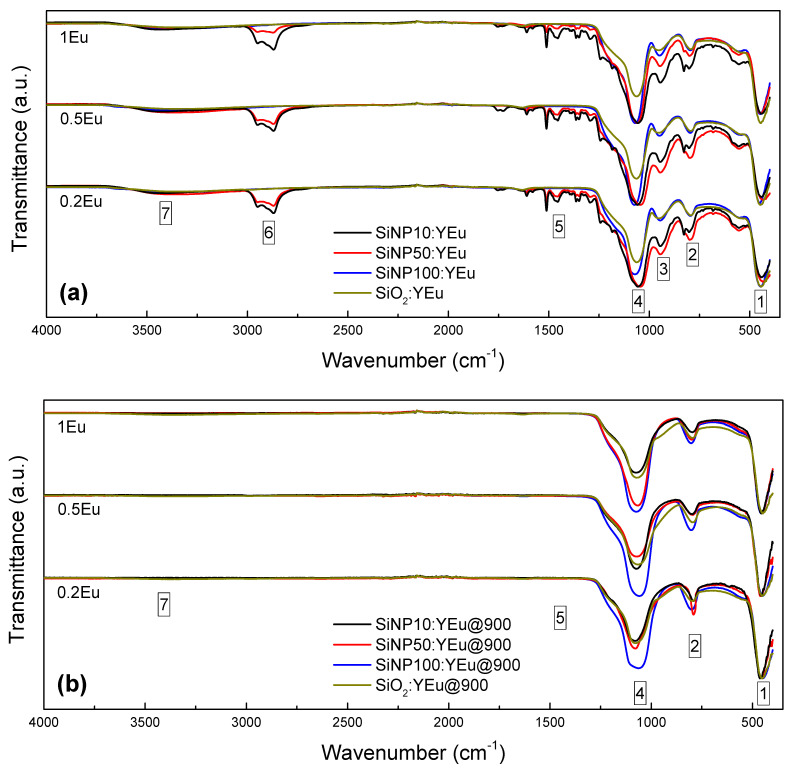
FT-IR spectra at room temperature of all SiNP: (**a**) before thermal treatment; and (**b**) after thermal treatment.

In addition, we recorded the FT-IR spectra of all studied samples (Figure 4). All the observed bands can be attributed to the SiO_2_ network and the presence of hydroxyl groups (–OH). A first comparison of the spectra of Eu-doped SiNP shows some slight differences in the intensity of certain spectra, which was due to the variation of the amount of material used during the FT-IR measurements.

Before and after thermal treatment, all SiNPs show the bands associated with the movement of oxygen in the SiO_2_ network: the swing “Peak 1” [22], symmetrical elongation “Peak 2” [22] and asymmetrical elongation “Peak 4” [22], as well as the shoulder at approximately 1200 cm^−1^, which is generally interpreted as an additional signature of the amorphous SiO_2_ network [17]. Thermal treatment causes displacement of the main peak at about 1100 cm^−1^ “Peak 4” to a higher wavenumber: 1059–1081, 1047–1078 and 1068–1079 cm^−1^ for **SiNP10:YEu**, **SiNP50:YEu** and **SiNP100:YEu**, respectively. This shift is explained by a decrease in the structural disorder of the amorphous silica with a variation of the bridging Si-O-Si bonding angle and a decreasing of the defects [18,19,20]. This decrease in disorder was also observed by DRX. In addition, we observed in all non-calcined samples the bands associated with the presence of OH groups. In addition, we can note that “Peak 3” corresponds to the symmetrical elongation of the Si-OH bond [22] “Peak 5” to the deformation of the OH bonds of the adsorbed water [22] and “Peak 7” to the overlap of the elongation peak of the OH hydrogen bond from water molecules adsorbed by the samples and the elongation peak of hydrogen from the silanol groups (Si-OH) within the glass [22].

Nevertheless, the SiNPs prepared by sol-gel in reverse microemulsion (10 and 50 nm) show an additional peak, “Peak 6”, between 2870 and 2950 cm^−1^. This peak is attributed to symmetrical and asymmetric elongation of CH_2_ and CH_3_ [30]. Moreover, “Peak 5” takes a new associated form with the bending of CH_3_ [30]. These hydrocarbon groups come mainly from the surfactant (Triton X-100), (Sigma Aldrich, Saint-Quentin Fallavier, France) co-surfactant (1-Hexanol) (Sigma Aldrich, Saint-Quentin Fallavier, France) and solvent (cyclohexane) used during the preparation of the reverse microemulsion. After thermal treatment at 900 °C, the peaks attributed to OH and the hydrocarbon groups decrease strongly or disappear, showing the strong reduction of these groups in the SiNP by thermal treatment. These results are confirmed by the elemental analyses of hydrogen. These analyses show 1.65, 4.63 and 1.81 wt.% for **SiNP10:1Eu**, **SiNP50:1Eu** and **SiNP100:1Eu**, respectively, and 0.14, 0.17 and 0.18 wt.% after the thermal treatment for the respective SiNP. Thus, we note a decrease of 91, 96 and 89 wt.% of hydrogen after calcination for **SiNP10:1Eu**, **SiNP50:1Eu** and **SiNP100:1Eu**, respectively. The large amount of hydrogen detected in **SiNP50:1Eu** is due to the use of a larger amount of surfactant, co-surfactant and solvent during the synthesis compared to that used for the preparation of **SiNP10:1Eu**. The origin of residual traces of hydrogen in the calcined samples could be due to water adsorbed from the air during the preparation of the various analyses [31].

**Figure 5:**
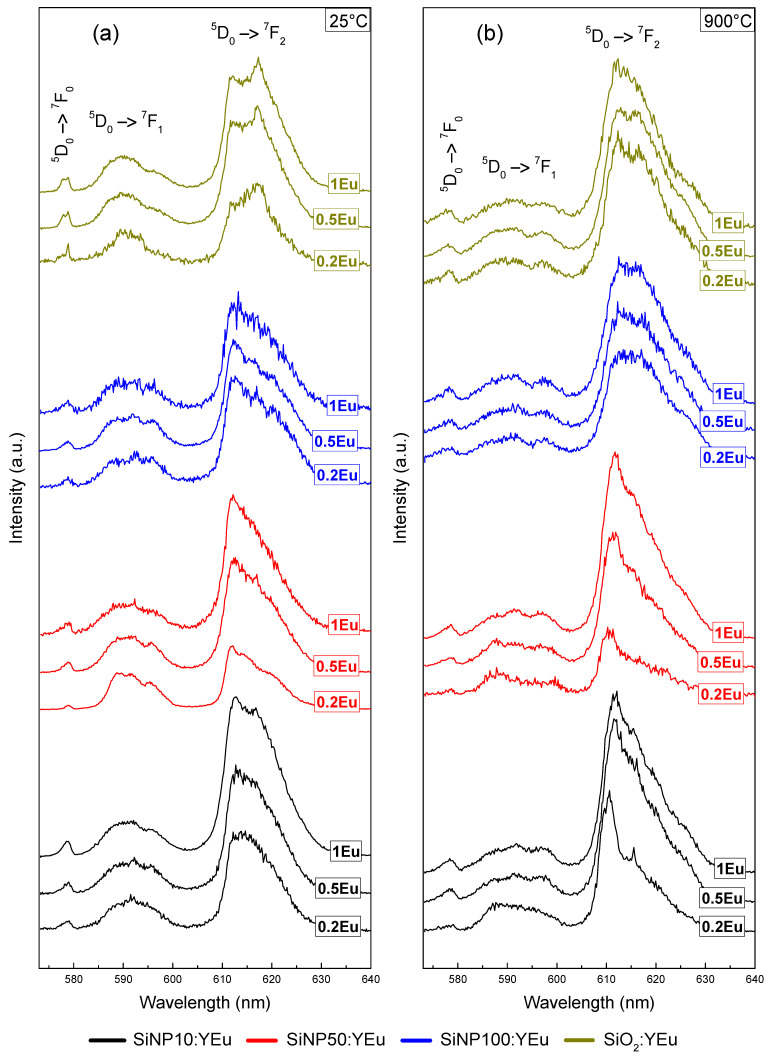
Emission spectra obtained under pulsed laser excitation at 532 nm at room temperature of Eu-doped silica nanoparticles and bulk (**a**) before and (**b**) after thermal treatment at 900 °C (the intensities are normalized to the maximum of the emission band ^5^D_0_ → ^7^F_1_).

To determine the effects of the different parameters (thermal treatment, Eu^3+^ concentration and nanoparticle size), emission spectra obtained under pulsed laser excitation at 532 nm are reported in Figure 5. All emission bands therein are characteristics of the radiative transitions of Eu^3+^ and are attributed to the ^5^D_0_ → ^7^F*_J_*_=0,1,…,6_ multiplet transitions [32]. The ^5^D_0_ → ^7^F_0_ transition, at shortest wavelength, is spin-forbidden and only allowed by *J*-mixing effects [32,33]. Both shape and peak wavelength are strongly influenced by the local electrostatic field around the luminescent ion. The nature of the second transition, ^5^D_0_ → ^7^F_1_, is mainly magnetic dipolar. The crystal field does not modify its intensity, but it influences splitting [32,34]. Information on the local environment around the rare-earth ions can be retrieved from the analysis of the ^5^D_0_ → ^7^F_1_ band shape [35]. The intensity of this band, insensitive to the local field, can be used to normalize and compare different emission spectra. The characteristics of the ^5^D_0_ → ^7^F_2_ transition (intensity and five-fold splitting) are extremely dependent on the crystal field strength [32,36]. The link between those features and the structure of the luminescent site is therefore trickier to analyze. For all Eu^3+^ concentrations investigated, the emission spectra of non-calcined SiNP are very similar, and they resemble those of Eu-doped silica bulk where inhomogeneous broadening is weak for emission bands ^5^D_0_ → ^7^F_0_ and ^5^D_0_ → ^7^F_1._ It is typically characteristic of the luminescent ion in an aqueous medium where the occupied sites are similar and mainly composed of OH groups [37].

Furthermore, the symmetry of the luminescent sites can be analyzed by comparing the intensities of the ^5^D_0_ → ^7^F_2_ and ^5^D_0_ → ^7^F_1_ transitions. Indeed, mixing of different parity states can occur when inversion symmetry is lowered, thus allowing electric dipolar transitions. Consequently, high symmetry sites are characterized by the low value of the peak intensity ratio R = I(^5^D_0_→^7^F_2_)/I(^5^D_0_→^7^F_1_), whereas different distorted sites are attributed to high value of *R*. The maximum intensity is considered to be the highest point of each peak. The obtained *R* values are presented in Table 3. The *R* values increase significantly with the amount of Eu for **SiNP10:YEu** and **SiNP50:YEu**. This is explained by a decrease in the symmetry of the sites occupied by the luminescent ions when their quantity increases [37,38]. In the bulk sample, variations of *R* are weak, and there is almost no variation of *R* for **SiNP100:YEu**. Since it is known that OH groups generate symmetrical aqueous environments for europium [39], increasing Eu^3+^ concentration seems to decrease the number of these aqueous environments for the smallest SiNP.

The size of the silica nanoparticles has only a slight impact on the emission spectra for the non-calcined samples, since spectra are globally very similar for the same doping concentration. However, one can observe slight differences between spectra of the **SiNP100:YEu** sample and the others: ^5^D_0_ → ^7^F_1_ and ^5^D_0_ → ^7^F_2_ bands present a more significant inhomogeneous broadening, which is an indication of a larger structural diversity of the luminescent sites in the largest SiNP. Considering the *R* intensity ratio, the lowest doping concentration would seem to be most affected by this trend.

Thermal treatment alters considerably the emission spectra for all samples. We can observe that the treatment leads to an inhomogeneous broadening accompanied by an increasing *R* ratio. This behavior is characteristic of solvent removal and is generally interpreted as a shift from an aqueous environment to a cation-dominated environment around the rare-earth ion: luminescent sites become more diverse and distorted with low symmetry [37,38]. Before the heat treatment, OH groups act as fluorescence-quenching centers due to energy transfer between Eu^3+^ and OH. The heat treatment removes the OH groups and improves the integration of Eu^3+^ ions in the glassy structure leading to a better fluorescence [40].

It can be assumed that the thermal treatment has a strong effect on the structure and/or the structural diversity of the luminescent sites. This reinforces previously discussed results: the IR spectra showing the elimination of water by calcination, as well as the elemental analysis showing hydrogen content decreasing by 91, 96 and 89 wt.% after calcination for **SiNP10:1Eu** (from 1.647 to 0.140 wt.%), **SiNP50:1Eu** (from 4.631 to 0.171 wt.%) and **SiNP100:1Eu** (from 1.811 to 0.184 wt.%), respectively. Moreover, the variation of the europium concentration has no influence other than that reported for the non-calcined samples.

**Figure 6:**
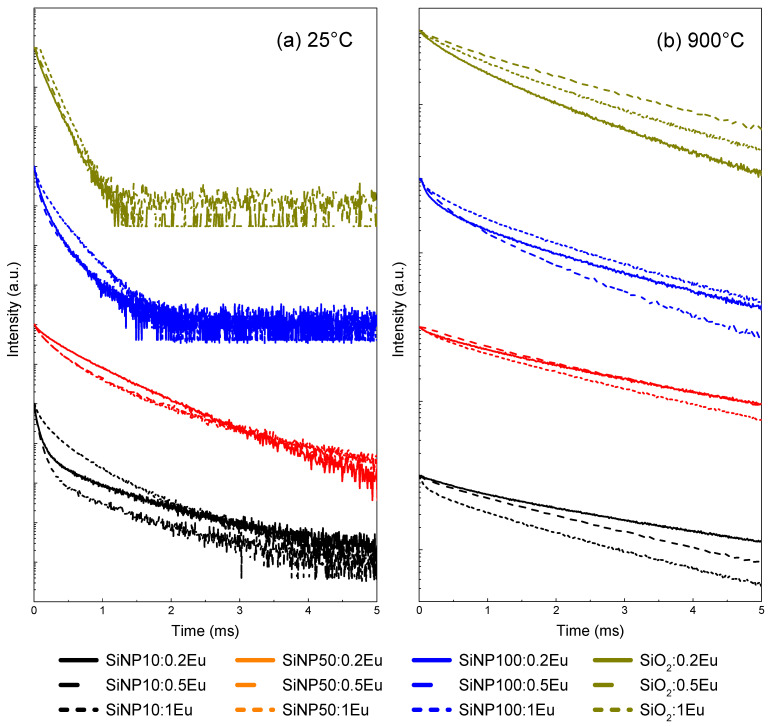
Luminescence decays of level ^5^D_0_ of Eu^3+^ measured at the maximum intensity of the emission band ^5^D_0_ → ^7^F_2_ in Eu-doped silica nanoparticles and bulk: (**a**) before thermal treatment at 900 °C; and (**b**) after thermal treatment at 900 °C.

Luminescence decay curves of the ^5^D_0_ level were recorded for all samples at the maximum intensity of the emission band ^5^D_0_ → ^7^F_2_. Figure 6 shows these decay curves before and after thermal treatment at 900 °C.

As in Eu-doped silica bulk [25], all samples show decays that are not simple exponentials. Rather than trying to fit these curves by means of several exponentials, which is difficult to interpret, we preferred to focus our attention on the comparison between samples in evaluating an average lifetime for each of them. This lifetime τ was therefore estimated according to the following Equation (1):

(1) τ=∫I(t)tdt/∫I(t)dt,

where I(t) is the intensity decay profile. The obtained values are reported in Table 4.

Aqueous environments and organic residues cause a quenching of the luminescence of Eu^3+^ ions, providing a non-radiative relaxation pathway [38]. In addition, residual hydroxyl groups and other organic groups can be eliminated by the thermal treatment, thereby increasing both the efficiency and decay time of luminescence. Therefore, by removing the quenching centers, the thermal treatment at 900 °C allows the lifetime for all samples to be increased. In the range of Eu^3+^ concentration investigated, the decay profiles and the average lifetimes are similar. This is in line with the observations made from the emission spectra and confirms that, up to 1 mol.%, there is no luminescence quenching by the effect of the concentration, which is generally attributed to the conjunction of a rare earth group with energy transfers between ions grouped by a mechanism of cross relaxation or a phonon-assisted energy transfer [41].

Before the thermal treatment, we notice that the average lifetimes are longer for **SiNP50:YEu** than for the other SiNP. The largest SiNP (100 nm) have the shortest τ, which are very close to those of the Eu-doped silica bulk. In **SiNP50:YEu,** the average lifetime τ decreases as the europium concentration increases, while no clear trend is observed in the smallest SiNP.

The influence of thermal treatment on the decay profiles is evident (Figure 6b), with a notable increase in lifetime for all samples. In addition, for **SiNP10:YEu** and **SiNP50:YEu**, the thermal treatment leads to similar lifetime values, which can be explained by the agglomerated and densified structures obtained after calcination. On the other hand, thermally treated **SiNP100:YEu** still have τ close to those of silica bulk, which are smaller than those of 10 and 50 nm SiNP.

## 5. Conclusions

We studied europium-doped silica nanoparticles prepared according to two sol-gel derived processes. Reverse microemulsion allows preparation of 10 and 50 nm particles, while 100 nm ones were prepared by the Stöber method. This article allows a direct comparison between the processes to be made, for the first time. For the structural characterizations, these SiNPs were characterized by BET, XRD and FT-IR. No significant effects of the amount of Eu^3+^ in silica nanoparticles on emission spectra or decays were observed. Before the heat treatment, luminescent properties of the nanoparticles prepared by reverse microemulsion are almost comparable but differ from the larger nanoparticles, which are similar to the silica bulk. The heat treatment at 900 °C reduces the aqueous environment around Eu ions and its fluorescence lifetime improves by one order of magnitude. This article demonstrates the potential of both sol-gel methods to prepare Eu-doped silica nanoparticles of controlled size. However, nanoparticles prepared with the reverse microemulsion process tend to melt when heated at high temperature. Future work will be devoted to improving the stability of these nanoparticles.

## Figures and Tables

**Table 1:** Starting compositions and nomenclatures of europium-doped silica nanoparticles.

Sample	Size (nm)	Amount of EuCl_3_.6H_2_O (mol %)
**SiNP10:0.2Eu**	10	0.2
**SiNP10:0.5Eu**	~10	0.5
**SiNP10:1Eu**	10	1
**SiNP50:0.2Eu**	50	0.2
**SiNP50:0.5Eu**	~50	0.5
**SiNP50:1Eu**	50	1
**SiNP100:0.2Eu**	100	0.2
**SiNP100:0.5Eu**	~100	0.5
**SiNP100:1Eu**	100	1

**Table 2:** Surface areas and pore volumes of **SiNPX:1Eu** and bulk **SiO_2_:1Eu** before and after thermal treatment at 900 °C.

Sample	BET Surface Area (m^2^/g)	Pore Volume (cm^3^/g)
**SiNP10:1Eu**	58	0.16
**SiNP10:1Eu@900**	41	0.22
**SiNP50:1Eu**	55	0.27
**SiNP50:1Eu@900**	41	0.23
**SiNP100:1Eu**	232	0.18
**SiNP100:1Eu@900**	26	0.16
**SiO_2_:1Eu**	334	0.32
**SiO_2_:1Eu@900**	213	0.09

**Table 3:** Intensity ratio R of Eu-doped silica nanoparticles and bulk before and after thermal treatment (T.T.) at 900 °C.

Sample	Y (%Eu)	R before T.T.	R after T.T.
	0.2	2.72	4.81
**SiNP10:YEu**	0.5	3.51	6.26
	1	4.35	6.21
	0.2	1.73	2.38
**SiNP50:YEu**	0.5	3.12	4.62
	1	3.82	6.35
	0.2	3.03	3.84
**SiNP100:YEu**	0.5	3.02	4.47
	1	3.31	5.01
	0.2	2.25	5.31
**SiO_2_:YEu**	0.5	3.34	5.09
	1	3.66	5.76

**Table 4:** Lifetimes of Eu-doped silica nanoparticles and bulk before and after thermal treatment (T.T.) at 900 °C.

Sample	Y (%Eu)	τ (ms) before T.T.	τ (ms) after T.T.
	0.2	0.26	4.81
**SiNP10:YEu**	0.5	0.15	6.26
	1	0.32	6.21
	0.2	0.45	2.38
**SiNP50:YEu**	0.5	0.39	4.62
	1	0.37	6.35
	0.2	0.11	3.84
**SiNP100:YEu**	0.5	0.11	4.47
	1	0.16	5.01
	0.2	0.11	5.31
**SiO_2_:YEu**	0.5	0.12	5.09
	1	0.13	5.76

## Data Availability

The data presented in this study are available on request.
